# Supplementary material for: Transcriptional Regulation and Gene Mapping of Internode Elongation and Late Budding in the Chinese Cabbage Mutant lcc
Source: Plants (Basel). 2024 Apr 12;13(8):1083. doi: 10.3390/plants13081083 (PMC11053886; doi:10.3390/plants13081083)
Supplement: Supplementary file 1 [file plants-13-01083-s001.zip › Table S1.pdf]

Table S1. Distribution of SNP markers on F_2 map.

Chromosome	Marker number	Length/cM
A01	134	103.420
A02	162	144.177
A03	241	174.437
A04	138	98.519
A05	178	128.047
A06	155	137.941
A07	157	125.375
A08	146	92.235
A09	206	144.413
A10	85	111.7
Total	1602	1260.271
