# Supplementary material for: Transcriptional Regulation and Gene Mapping of Internode Elongation and Late Budding in the Chinese Cabbage Mutant lcc
Source: Plants (Basel). 2024 Apr 12;13(8):1083. doi: 10.3390/plants13081083 (PMC11053886; doi:10.3390/plants13081083)
Supplement: Supplementary file 1 [file plants-13-01083-s001.zip › Table S3.pdf]

Table S3. DEGs (differentially expressed genes) between *lcc* and R500 in the hormone regulatory pathway at seedling stage.

Gene ID	Gene name
BraA03g023790.3C	AGL20
BraA02g003340.3C	AGL25,FLC
BraA03g037500.3C	AHP4
BraA03g021580.3C	ARR16
BraA07g026930.3C	AUX1, LAX
BraA07g006070.3C	BAK1,SERK4
BraA08g028240.3C	BZR2
BraA05g001030.3C	CCA1
BraA02g043680.3C	CDF1
BraA02g005190.3C	CHS-1
BraA03g005990.3C	CHS-2
BraA10g024990.3C	CHS-3
BraA10g002940.3C	CRY2
BraA07g031650.3C	TSF
BraA03g002880.3C	GA20OX3-1
BraA10g030020.3C	GA20OX3-2
BraA10g001200.3C	GA2OX6
BraA03g013900.3C	GH3.6
BraA09g040290.3C	GI
BraA05g040050.3C	GID1a
BraA09g052310.3C	GID1b
BraA02g003870.3C	HY5-1
BraA05g029990.3C	HY5-2
BraA08g010620.3C	IAA14
BraA04g032040.3C	JAR1-1
BraA05g001430.3C	JAR1-2
BraA02g021020.3C	JAZ
BraA03g005650.3C	JAZ
BraA05g029670.3C	JAZ
BraA06g015450.3C	ARF5-1
BraA08g028060.3C	ARF5-2
BraA04g030640.3C	PIF4
BraA04g032300.3C	PRR9-1
BraA05g001070.3C	PRR9-2
BraA09g006460.3C	RFWD2, COP1
BraA02g007410.3C	SAUR
BraA02g007430.3C	SAUR
BraA02g007480.3C	SAUR
BraA02g007500.3C	SAUR
BraA02g035550.3C	SAUR
BraA03g008240.3C	SAUR
BraA03g008290.3C	SAUR
BraA03g008320.3C	SAUR
BraA07g002710.3C	SAUR
BraA10g011930.3C	SAUR
BraA03g024140.3C	SPA1
BraA01g007440.3C	TCH4
BraA08g018140.3C	TCH4
BraA03g044360.3C	TOC1, APRR1
