# Supplementary figures and images for: Transcriptional Regulation and Gene Mapping of Internode Elongation and Late Budding in the Chinese Cabbage Mutant lcc
Source: Plants (Basel). 2024 Apr 12;13(8):1083. doi: 10.3390/plants13081083 (PMC11053886; doi:10.3390/plants13081083)

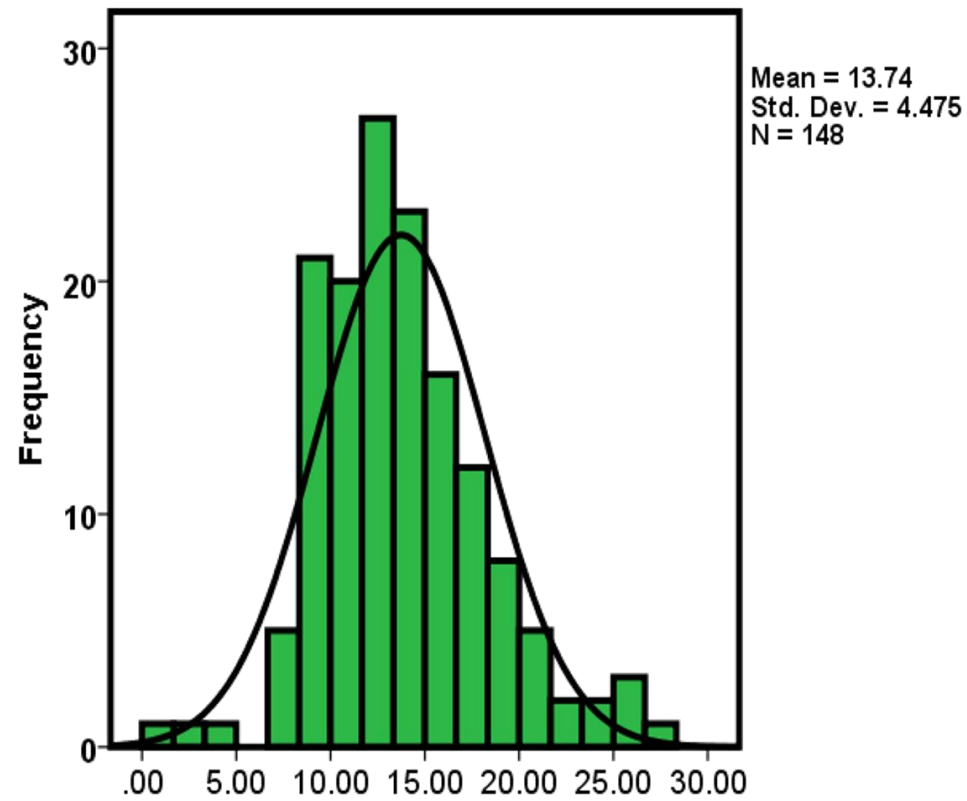

**Figure S1. Distribution of hypocotyl length in F<sub>2</sub> population.**

Supplement: Supplementary file 1 [file plants-13-01083-s001.zip › Figure S1.pdf]

# A01-A10

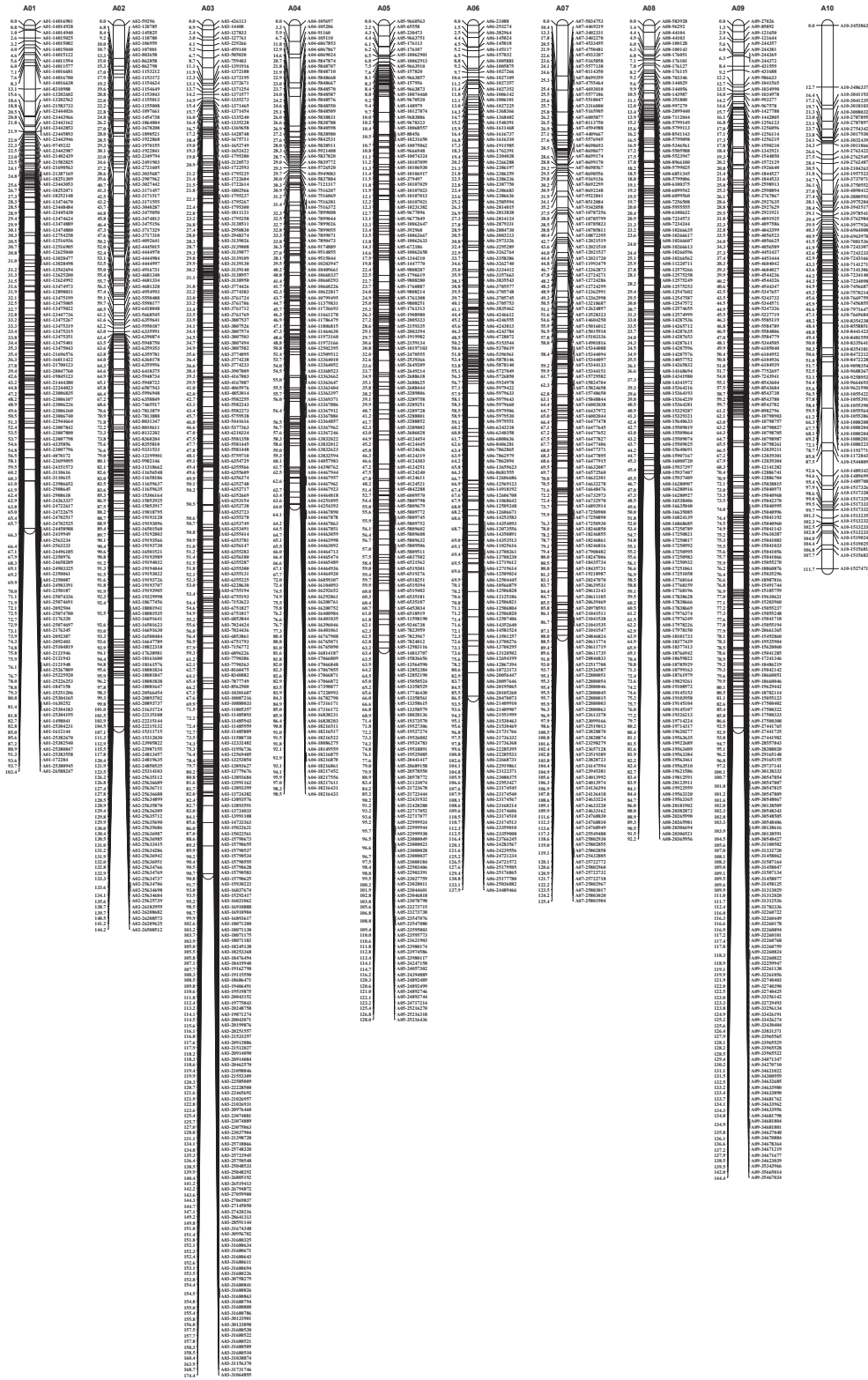

Figure S2. Distribution of SNP markers on genetic linkage map.

Supplement: Supplementary file 1 [file plants-13-01083-s001.zip › Figure S2.pdf]
